# Artificial intelligence for non-mass breast lesions detection and classification on ultrasound images: a comparative study

**DOI:** 10.1186/s12911-023-02277-2

**Published:** 2023-09-04

**Authors:** Guoqiu Li, Hongtian Tian, Huaiyu Wu, Zhibin Huang, Keen Yang, Jian Li, Yuwei Luo, Siyuan Shi, Chen Cui, Jinfeng Xu, Fajin Dong

**Affiliations:** 1 Jinan University, Guangzhou, Guangdong 510632 China; 2 Ultrasound Department, Shenzhen People’s Hospital (The Second Clinical Medical College, Jinan University), Shenzhen, Guangdong 518020 China; 3 Department of Thyroid and Breast Surgery, Shenzhen People’s Hospital (The Second Clinical Medical College, Jinan University), Shenzhen, Guangdong 518020 China; 4 Research and development department, Illuminate, LLC, Shenzhen, Guangdong 518000 China

**Keywords:** Non-mass breast lesions, Artificial intelligence, Ultrasound

## Abstract

**Background:**

This retrospective study aims to validate the effectiveness of artificial intelligence (AI) to detect and classify non-mass breast lesions (NMLs) on ultrasound (US) images.

**Methods:**

A total of 228 patients with NMLs and 596 volunteers without breast lesions on US images were enrolled in the study from January 2020 to December 2022. The pathological results served as the gold standard for NMLs. Two AI models were developed to accurately detect and classify NMLs on US images, including DenseNet121_448 and MobileNet_448. To evaluate and compare the diagnostic performance of AI models, the area under the curve (AUC), accuracy, specificity and sensitivity was employed.

**Results:**

A total of 228 NMLs patients confirmed by postoperative pathology with 870 US images and 596 volunteers with 1003 US images were enrolled. In the detection experiment, the MobileNet_448 achieved the good performance in the testing set, with the AUC, accuracy, sensitivity, and specificity were 0.999 (95%CI: 0.997-1.000),96.5%,96.9% and 96.1%, respectively. It was no statistically significant compared to DenseNet121_448. In the classification experiment, the MobileNet_448 model achieved the highest diagnostic performance in the testing set, with the AUC, accuracy, sensitivity, and specificity were 0.837 (95%CI: 0.990-1.000), 70.5%, 80.3% and 74.6%, respectively.

**Conclusions:**

This study suggests that the AI models, particularly MobileNet_448, can effectively detect and classify NMLs in US images. This technique has the potential to improve early diagnostic accuracy for NMLs.

## Background

Breast tumors can present as either as mass breast lesions (MLs) or non-mass breast lesions (NMLs) [1]. NMLs manifest as confined asymmetry on two orthogonal planes without conspicuous margins or shapes, which fails to meet the strict criteria of “mass” defined by BI-RADS [2]. And it is a rare form of breast lesions, occurring in only 9.2% of all cases and less commonly than ML lesions [3]. Moreover, conventional US images show a lack of clear boundaries on both planes for NMLs, making them more difficult to identify than MLs [4]. Thus, accurate diagnose of NMLs is clinically important and a challenging task.

Currently, Mammography, Ultrasound (US) and Magnetic Resonance Imaging (MRI) are three valuable tools used in the early screening for breast tumors [5–7]. When selecting the optimal screening tools for breast tumors, there are many factors to consider, including patient age, breast density, and the presence of any symptoms [8]. Compared to other screening tools, US offers several advantages, such as lower cost, no ionizing radiation, and assessing images in real-time, especially suitable for Asian dense breast women [9, 10]. Though US is a valuable tool for diagnosing NMLs, it is not without its limitations. Accurately diagnosing NMLs using US can be challenging due to several factors, including the quality of the US equipment and the experience of the radiologist, as well as the lesion’s size and location [11]. In addition, given the overlapping between benign and malignant features of NMLs, it is difficult make a further accurate diagnosis [12–15]. Therefore, it is necessary to avoid missed diagnosis and improve the precision of diagnosis with a novel method.

Artificial intelligence (AI), a computer science subfield, has made a great breakthrough in the image recognition task. And medical image from routine clinical process is an important research field of AI [16]. Recently, the application of AI within the field of imaging diagnostics has led to remarkable achievements across a range of subfields, such as the diagnosis of lung cancer [17], skin cancer [18],and breast cancer [19]. Especially for breast cancer diagnosis, there are numerous studies reported an AI system achieved a superior breast radiologists’ level in diagnosing breast tumor [20]. However, these AI model mainly focused on MLs, while study on NMLs is rarely. Furthermore, whether AI model can help radiologist detect and classify NMLs is still to explore.

Therefore, this study proposed an AI model trained on MobileNet and DenseNet121 to detect and diagnose NMLs on US images. It is divided into two steps: firstly, to develop the AI model for detecting NMLs on the normal breast US images, and secondly, according to the prior works in AI for breast mass tumor [21], to investigate the efficacy of AI models trained on MLs US images to diagnosing the benign or malignant NMLs.

## Methods

### Patients

**Fig. 1:**
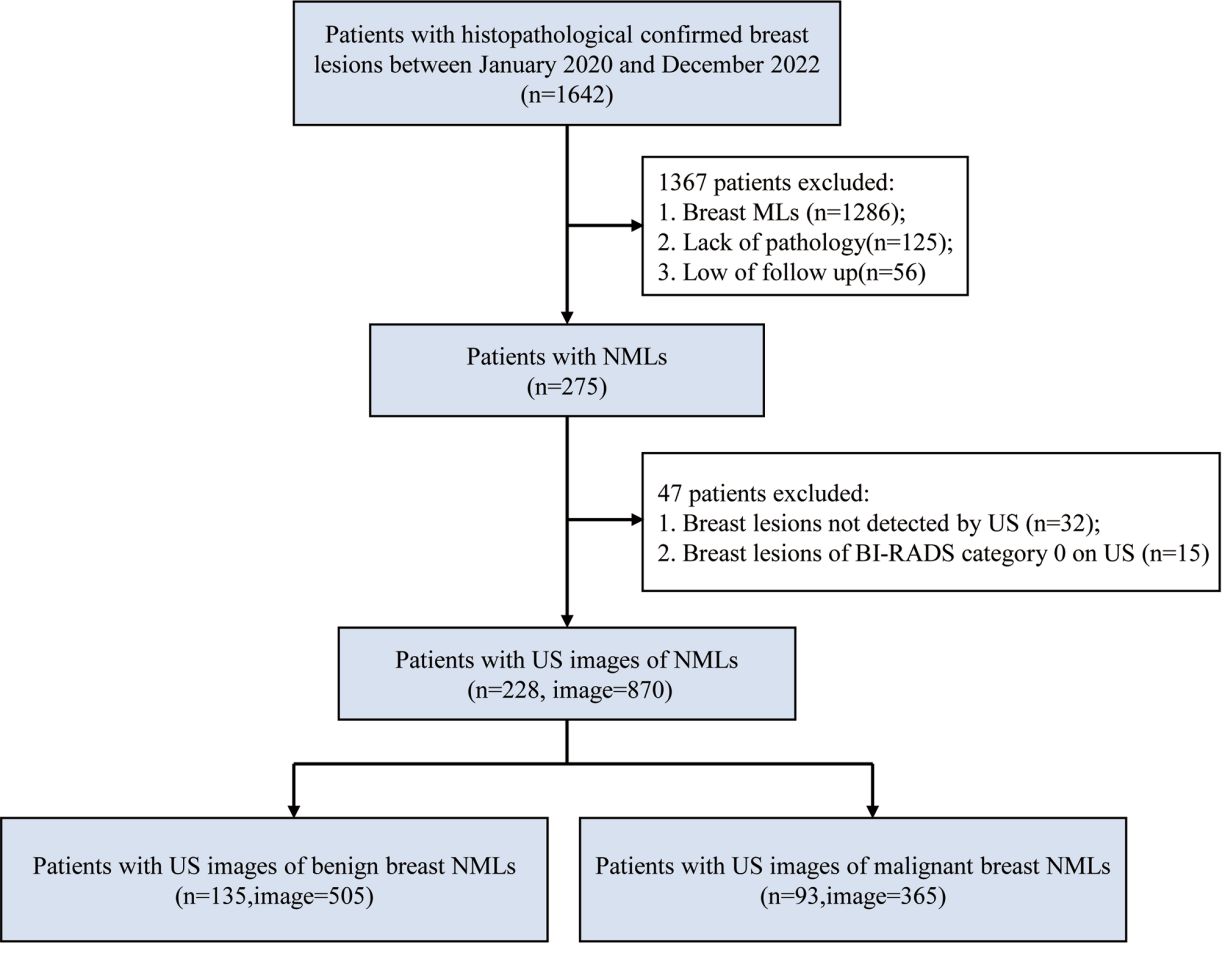
Flow chart of NMLs patient’s selection

This retrospective study was approved by the Institutional Review Board of the Shenzhen People’s Hospital, specifically the Medical Ethics Committee of Shenzhen People ‘s Hospital. Informed consent was waived by the same ethics committee that approved the study. Consecutive patients at Shenzhen People’s Hospital from January 2020 to December 2022 were enrolled (Fig. 1). The inclusion criteria were: (a) Patients who received breast US examination and were found to have breast lesions.(b)The breast lesions on the US images were consistent with the features of NMLs as described in the literature by Choe et al [22]. (c) The NMLs were diagnosed as benign or malignant according pathology analysis or follow-up exceeding 2 years. The exclusion criteria were: (a) Breast MLs. (b) Lack of pathology. (c) Loss of follow up. (d) Breast lesions not detected by US. (e) Breast lesions of BI-RADS category 0 on US. In addition, female volunteers were included for breast US examination to obtain normal breast US images.

### Data acquisition and processing

The US images were acquired with different equipment, including Mindray Resona 7 (Mindray, China, equipped with L11-3U linear array transducer)、Phillips EPIQ5 (Philips, The Netherlands, equipped with L12-5 linear array transducer and GE LOGIQ E9 (GE, USA, equipped with ML6-15-D linear array transducer) by two radiologists with 15 and 10 years of experience in breast US examinations. The US images were exported from the equipment as JPEG images.

In our previous work, we had collected MLs US images to develop AI model to diagnose breast mass tumor [21]. Based on the inclusion and exclusion criteria of MLs, 4988 patients with 13,247 MLs US images were finally included. In this study, we selected MLs US images as the data set to train the AI model.

The field-of-view (FOV) is obtained from the original US image removing device and patient-related information. Subsequently, the FOV are transformed into squares and scaled to 448 × 448, thus serving as the model training and testing data.

### AI model

**Fig. 2:**
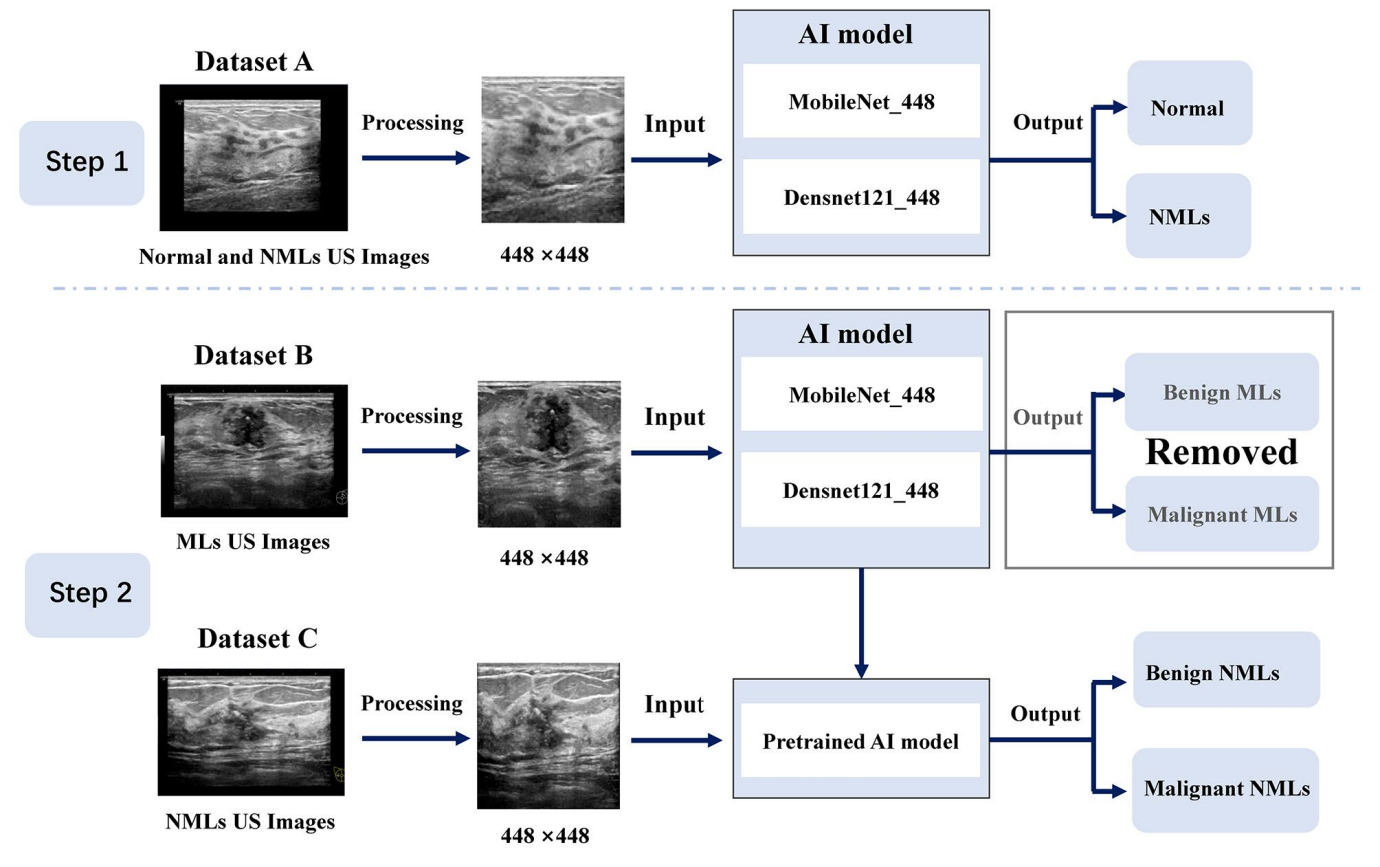
Proposed network scheme of the AI model for detecting and classifying NMLs

we utilized the current mainstream AI models, which included MobileNet (a lightweight convolutional neural network) and DenseNet121 (a well-known deep learning model with fewer parameters) to develop AI models in this study (Fig. 2). In order to differentiate them from other models, we named MobileNet and DenseNet121 based on the image size of input data as MobileNet_448 and DenseNet121_448, respectively.

### Detection experiment

To select the optimal AI model for NMLs detection in the normal breast US images, we proposed training both MobileNet_448 and DenseNet121_448 in the dataset A (including normal breast US images and NMLs US images). First, the dataset A was split into training, validation and testing set based on a ratio of 7:1:2. Second, we developed AI model with the default setting of training 300 epochs, while setting Early Stopping, 15 epochs of validation set loss does not drop will end the training early. The batch size was set to 16, using Focal Loss as the loss function, and Adam as the optimizer with a learning rate of 0.001. Finally, we evaluated the performance of both models in the testing set to determine which one is better suited for NMLs detection experiment.

### Classification experiment

For the classification experiment to diagnose benign and malignant NMLs, we developed AI model with MobileNet_448 and DenseNet121_448 in the dataset B (including MLs US images), similarly. Initially, the MLs US images in the dataset B was split into training, validation and internal testing set based on a ratio of 8:1:1. In addition, the dataset C (including NMLs US images) served as external testing set. Second, we developed AI model with the default setting of training 300 epochs, while setting Early Stopping, 15 epochs of validation set loss does not drop will end the training early. The batch size was set to 16, using Focal Loss as the loss function, and Adam as the optimizer with a learning rate of 0.001. To enhance model performance, we included a learning rate decay strategy: if the validation set loss didn’t decrease for 5 consecutive epochs, the learning rate was reduced to 1/10 of its original value.

The performance of AI model pretrained with MLs US images was evaluated in the internal testing set. Ultimately, we validated the diagnostic effectiveness of developed AI model for NMLs in external testing set. The Grad-CAM technique was utilized to explain how the optimal AI model discriminates benign and malignant NMLs.

### Statistical analysis

Expression of continuous variable data is achieved using mean ± standard deviation. Expression of categorical variable data is achieved using percentage. Within-group differences were compared using the paired sample t-test. Statistical analysis was performed using R version 4.2.2 (R Core Team, 2021). Draw the receiver operating characteristic (ROC) curve, compute the area under the curve (AUC), and output the 95% confidence interval (95% CI). And then output the cut-off value, specificity, sensitivity, and accuracy. Statistical significance is determined at a p-value of less than 0.05.

## Results

### Clinical characteristics

In this study, 228 NMLs patients were included. The analyses of clinical characteristics of the NMLs patients are presented in Table 1. The clinical characteristics between malignant and benign NMLs patients did not show any statistically significant difference (p > 0.05).

**Table 1:** Clinical characteristics of patients with NMLs

Parameter	Malignant(n = 93)	Benign (n = 135)	p value
Mean Age ± SD(y)	46.21 ± 8.53	45.32 ± 9.26	>0.05
Mean BMI ± SD (kg/m^2^)	22.16 ± 2.09	21.93 ± 1.98	>0.05
Family history of breast cancer			>0.05
Yes	4	0	
No	89	135	
Breast pain			>0.05
Yes	37	59	
No	56	76	
Nipple discharge			>0.05
Yes	0	0	
No	93	135	
Palpable mass			>0.05
Yes	0	0	
No	93	135	
Architectural changes			>0.05
Skin thickening	0	0	
Architectural distortion	0	0	
No change	93	135	
Position of lesions			
Left	39	73	
Right	54	62	
Enlarged axillary lymph nodes			>0.05
Yes	3	0	
No	90	135	

*Note: BMI: Body mass index. p value: Comparison between Malignant and Benign*

In the dataset, 870 NMLs US images from NMLs patients,13,247 MLs US images from 3,447 MLs patients and 1003 normal breast US images from 596 volunteers were included. The distribution of the dataset in the detection and classification experiment are summarized in Table 2. In the dataset A, the training, validation, and internal testing sets contain 1310, 188, and 375 US images, respectively. In the dataset B, the training, validation, and internal testing set contain 10,619, 1289, and 1339 US images, respectively. The dataset C was inputted as external testing set containing 870 US images.

**Table 2:** The distribution of the dataset in the detection and classification experiment

Dataset		Pathology		Total
**Dataset A**		**Normal**	**NMLs**	
Training Set	Patients, n, %	481	172	653
		60%	40%	
	Images, n, %	702	608	1310
		54%	46%	
Validation Set	Patients, n, %	66	31	97
		68%	32%	
	Images, n, %	100	88	188
		53%	47%	
Testing Set	Patients, n, %	49	25	74
		66%	34%	
	Images, n, %	201	174	375
		54%	46%	
**Dataset B**		**Benign MLs**	**Malignant MLs**	
Training Set	Patients, n, %	2728	1249	3977
		69%	31%	
	Images, n, %	7260	3359	10,619
		68%	32%	
Validation Set	Patients, n, %	352	158	510
		69%	31%	
	Images, n, %	863	426	1289
		67%	33%	
Internal Testing Set	Patients, n, %	338	163	501
		67%	33%	
	Images, n, %	862	477	1339
		64%	36%	
**Dataset C**		**Benign NMLs**	**Malignant NMLs**	
External Testing Set	Patients, n, %	135	93	228
		59%	41%	
	Images, n, %	505	365	870
		58%	42%	

### Detection performance of NMLs

**Fig. 3:**
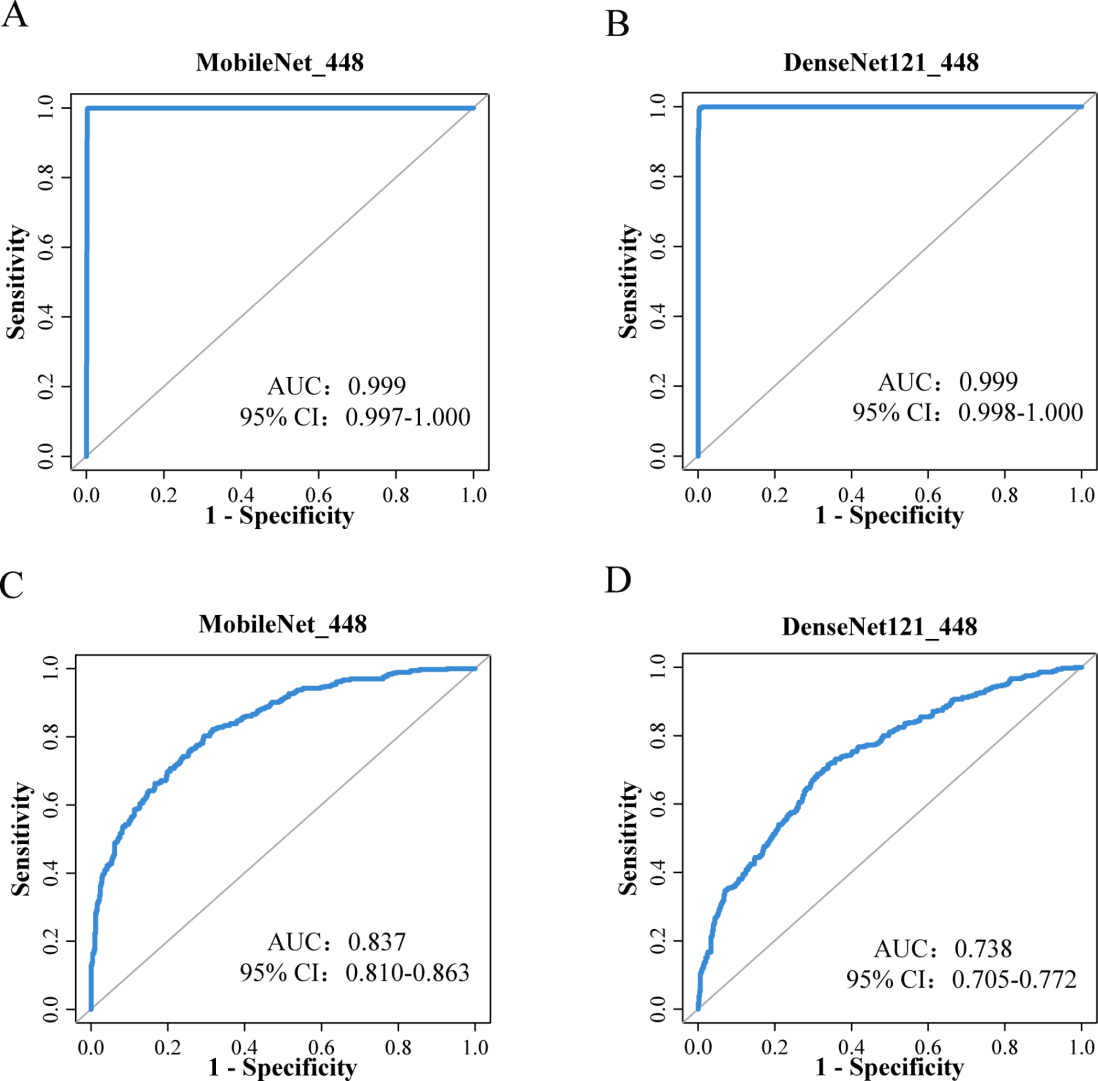
ROC of AI model in the testing set. Note: AUC: area under the curve, 95% CI: 95% Confidence Interval. (**A**) and (**B**) is the ROC of AI model in detection experiment. (**C**) and (**D**) is the ROC of AI model in classification experiment

The testing set results for the AI models are presented in Table 3. MobileNet_448 achieved the performance with an AUC of 0.999(95%CI: 0.997-1.000) along with accuracy, sensitivity and specificity of 96.5%,96.9% and 96.1%, respectively. DenseNet121_448 achieved the performance with an AUC of 0.999(95%CI: 0.998-1.000) and an accuracy, sensitivity and specificity of 96.5%,96.9% and 96.1%, respectively. There is no statistically in the AUC between MobileNet_448 and DenseNet121_448 (p>0.05). Figure 3(A and B) is the ROC curve of MobileNet_448 and DenseNet121_448 in the testing set.

**Table 3:** Performance of the AI models for the testing set

AI Model	AUC (95%CI)	Cut-off	Sensitivity(%)	Specificity(%)	Accuracy (%)	p value
Detection						
MobileNet_448	0.999(0.997-1.000)	0.985	99.9	99.7	99.8	Na
DenseNet121_448	0.999(0.998-1.000)	0.676	99.7	99.6	99.6	>0.05
Classification						
MobileNet_448	0.837(0.810–0.863)	0.489	70.5	80.3	74.6	Na
DenseNet121_448	0.738(0.705–0.771)	0.441	68.8	68.9	68.9	<0.05

*Note: p value: Comparison of MobileNet_448 with DenseNet121_448*

### Diagnostic performance of NMLs

The testing set results for the AI models are presented in Table 3. MobileNet_448 exhibited optimal diagnostic performance, achieving an AUC of 0.837 (95%CI: 0.810–0.863) and demonstrating an accuracy, sensitivity, and specificity of 68.8%, 68.9%, and 68.9%, respectively. Figure 3(C and D) is the ROC of MobileNet_448 and DenseNet121_448 in the external testing set.

## Disscusion

Currently, US is commonly utilized as a primary screening approach for NMLs. However, the absence of international standards, such as BI-RADS [2], has potentially led to missed identification and impacted the precision of diagnoses of NMLs. In this study, we developed an AI model that includes a lightweight and efficient neural network (MobileNet_448) and a dense and heavily connected neural network (DenseNet121_448). The model was trained with US images to detect and evaluate benign and malignant NMLs. In the detection experiment, MobileNet_448 showed promising performance in discriminating NMLs in the testing set. It was no difference in AUC (0.999) compared to DenseNet121_448. In the classification experiment, MobileNet_448 achieved an AUC of 0.837 in diagnosing benign and malignant NMLs in the testing set, which exceeded DenseNet121_448’s AUC (0.738).

Early detection is the first step for diagnosing NMLs. Several studies have indicated that deep learning’s performance in the detection of NMLs could match that of expert radiologists [23, 24]. O. Hadad et al. used a cross-modal deep learning for breast MRI mass/non-mass lesions classification task. The cross-modal learning was achieved with accuracy of 0.94 and AUC of 0.98 [23]. Fernando Soares, et al. proposed the use of a support vector machine to differentiate and categorize regions from mammograms into either mass or non-mass. The classification of MLs and NMLs using the proposed methodology yielded an average accuracy of 98.88% [24]. These studies were consistent with our findings. However, some differences between the studies were noted. We validated that AI model can detect NMLs from normal breast US images with AUC of 0.999 that compared with previously reported in MRI and Mammograms. Thus, by using MobileNet_448 or DenseNet121_448 to identify potential areas of concern in US images, US radiologists can focus their attention on those areas and make more informed diagnoses. Ultimately, this can lead to better patient outcomes.

**Fig. 4:**
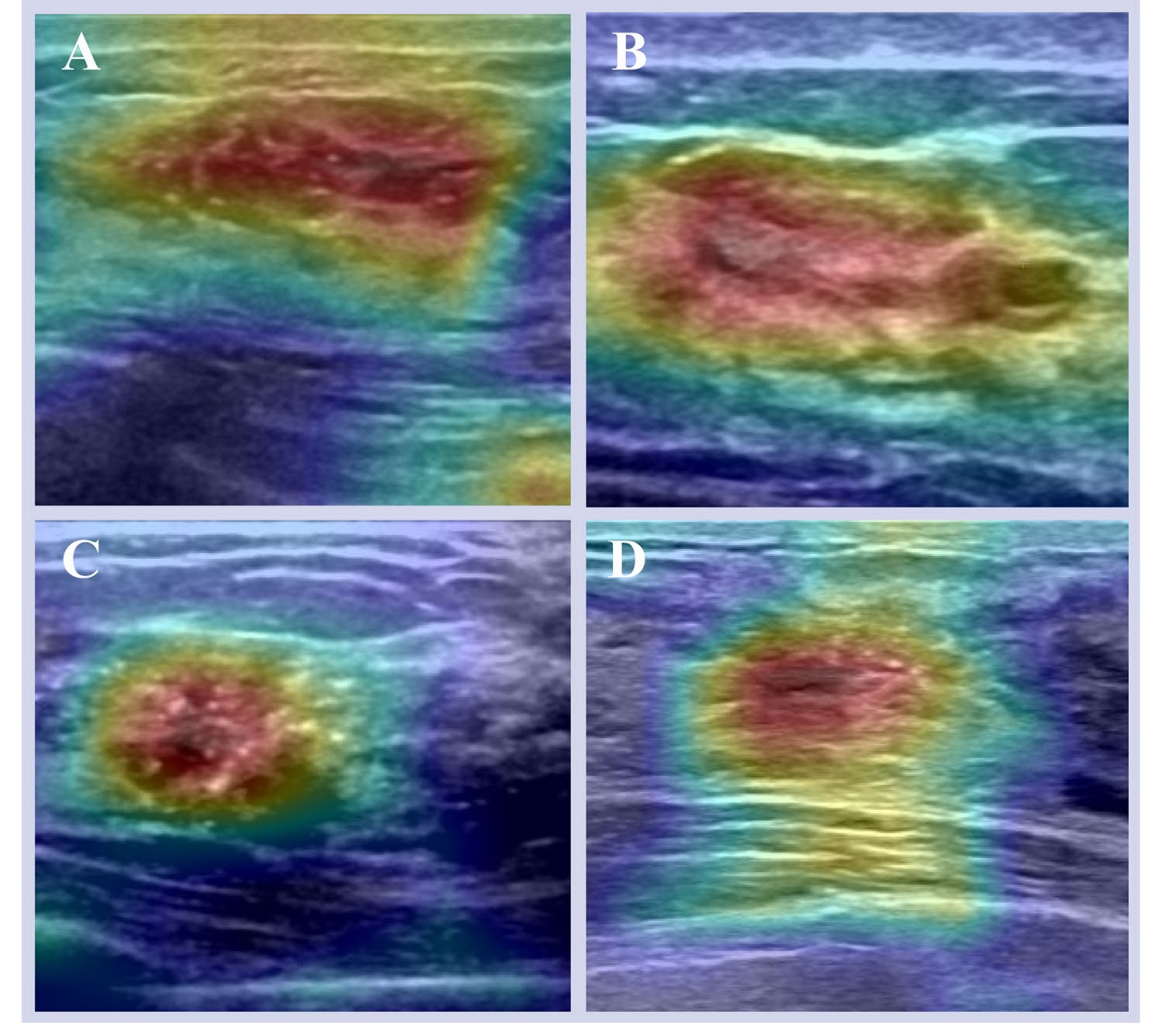
Grad-CAM plots explained how the MobileNet_448 model discriminates benign and maglinant NMLs.

In clinical practice, considerable overlap exists between the conventional B-mode US features of malignant NMLs and those of benign NMLs, such as fibrocystic change, sclerosing adenosis, atypical ductal hyperplasia, and intraductal papilloma [12, 25, 26]. Correct identification is a challenging task that frequently results in missed diagnoses or misdiagnoses. In this study, the MobileNet_448 trained on MLs US images yielded an AUC of 0.837 and accuracy of 74.6% in the classification of benign and malignant NMLs in the testing set. These results indicated that the MobileNet_448 model can effectively differentiate between benign and malignant NMLs using US imaging. And the regions of interest that the models focus on for NMLs detection and classification is the interior of lesions (Fig. 4). Besides, the MobileNet_448 model obtained an AUC that was either equal to or higher than previous studies. M Lin, et al. investigated the positive predictive value of classification of NMLs on US images following BI-RADS [27]. Setting BI-RADS 4B as the threshold, the AUC was 0.62 in their research. Choi J S, et al. proposed a way to evaluate NMLs utilizing a combination of shear-wave elastography and color Doppler US. It can achieve an AUC of 0.801, indicating that the inclusion of additional information regarding the elasticity and vascularity of breast NMLs can improve the diagnostic performance [28]. Therefore, the MobileNet448 model has the potential to be a reliable tool to diagnose NMLs.

Moreover, this study also demonstrated that the MobileNet model is particularly effective in diagnosing breast tumors, which is consistent with previous research reports [3, 29–31]. In the task of diagnosing benign and malignant NMLs, the MobileNet_448 can effectively diagnose NMLs with the AUC of 0.837 and ACC of 0.746 that outperformed DenseNet121_448. We suggested that some common features in US images used to identify malignant breast lesions, which are seen in both MLs and NMLs. And these images features can be learned by MobileNet_448 to distinguish benign and malignant NMLs. Therefore, the MobileNet_448 model may provide a more accurate and reliable method for diagnosing NMLs, potentially leading to better patient outcomes.

However, this study has several limitations. First, we conducted the research with a limited sample size which included 228 patients and 228 NMLs. To address this limitation, a larger population must be included in a prospective study. In addition, only 2D grayscale images were included in the study, which potentially can misrepresent the US images characteristics of NMLs. We will include multimodality (color Doppler flow imaging, pulse-wave Doppler US, contrast enhanced US) in the future.

## Conclusion

In this study, the MobileNet_448 and DenseNet121_448 we developed can effectively detect and classify NMLs on US images. By comparing, DenseNet121_448 has shown optimal performance in the early screening stages of NMLs, making it a valuable tool for assisting US radiologists in the future to better diagnose NMLs.

## Data Availability

The datasets used and analyzed during the current study are available from the corresponding author on reasonable request.

## Abbreviations

NMLs: Non-mass breast lesions
MLs: Mass breast lesions
AI: Artificial intelligence
US: Ultrasound
MRI: Magnetic Resonance Imaging
CI: Confidence interval
AUC: Area under the receiver operating characteristic curve
